# Lower albumin levels are associated with frailty measures, trace elements, and an inflammation marker in a cross-sectional study in Tanushimaru

**DOI:** 10.1186/s12199-021-00946-0

**Published:** 2021-02-19

**Authors:** Maki Yamamoto, Hisashi Adachi, Mika Enomoto, Ako Fukami, Sachiko Nakamura, Yume Nohara, Akiko Sakaue, Nagisa Morikawa, Hitoshi Hamamura, Kenta Toyomasu, Yoshihiro Fukumoto

**Affiliations:** 1 Division of Cardio-Vascular Medicine, Kurume University School of Medicine, Kurume, Japan; 2 Department of Community Medicine, Kurume University School of Medicine, 67 Asahi-machi, Kurume, 830-0011 Japan

**Keywords:** Albumin, Frailty measures, Cognitive function, Trace elements, Epidemiology

## Abstract

**Background:**

There is little data on the association between the lower nutrition represented by serum albumin levels and related factors in a general population. The present study aimed to determine whether the albumin level positioned as some kind of biomarker with frailty measures, trace elements, and an inflammation marker.

**Methods:**

In 2018, we performed an epidemiological survey in 1368 subjects who resided in Tanushimaru, Japan, in which we examined the blood chemistry including albumin, trace elements, hormone levels, and carotid ultrasonography. Albumin levels were categorized into 4 groups (G1 [3.2–3.9 mg/dL], G2 [4.0–4.3 mg/dL], G3 [4.4–4.6 mg/dL], and G4 [4.7–5.3 mg/dL]). The participants underwent measurements of handgrip strength and were tested by asking to walk 5 m. Their cognitive functions were evaluated by the mini-mental state examination (MMSE).

**Results:**

Multiple stepwise regression analysis demonstrated that albumin levels were significantly and independently associated with age (inversely), systolic blood pressures, estimated glomerular filtration rate (eGFR), MMSE score, frailty measures (handgrip strength), an inflammation marker (high-sensitivity C-reactive protein), hormones (growth hormone (inversely) and insulin-like growth factor-1), and trace elements (calcium, magnesium, iron, and zinc), with a linear trend.

**Conclusions:**

Lower albumin levels, even in the normal range, were found to be related factors of frailty measures, trace elements, and an inflammation marker in a general population.

## Background

It has been reported that hypoalbuminemia is associated with greater all-cause mortality in a general population [1, 2]. We have also reported the impact of lower albumin levels on long-term mortality in our cohort [3]. The normal serum concentration of albumin in healthy adults is ≥ 4.0 g/dL, while hypoalbuminemia is defined as a serum albumin level of ≤ 3.4 g/L [4]. A meta-analysis by Vincent et al. [5] suggested that hypoalbuminemia is an independent risk factor in patients with acute illness. Hypoalbuminemia was associated with frailty [6], cognitive impairment [7], and trace elements deficiency [8]. It was demonstrated that low serum albumin is a risk factor for frailty in elderly people with diabetes in a Japanese cross-sectional study [9]. In the longitudinal study, lower levels of serum albumin are associated with future decline in functional performance in older people [10]. However, most reports have been limited to older subjects [6, 7] and to older hospital patients [8]. The age of the target cases in previous papers [6, 7] was over 60 years old.

Reports with association between hypoalbuminemia and growth hormone (GH) or insulin-like growth factor-1 (IGF-1) were also scant. A study of hemodialysis patients revealed that hypoalbuminemia was significantly associated with lower IGF-1 [11]. In the setting of malnutrition, GH was positively, and IGF-1 was negatively, associated with inflammation markers such as high-sensitivity C-reactive protein (Hs-CRP) [12], suggesting that inflammatory markers were independent predictors of frailty [13].

The above-mentioned studies were all clinical and dealt with older adults and patients. Therefore, we aimed to determine whether lower albumin levels (< 4.0 mg/dL) are associated with frailty measures, trace elements, an inflammation marker, and nutrition intake in a large number of healthy subjects from a Japanese general population in the present study.

## Methods

### Subjects

We enrolled subjects from the typical farming town, Tanushimaru, located in Kyushu, the southwestern island of Japan conducted in 2018 as a Japanese cohort of the Seven Countries Study [14]. The subjects of this study are 1368 participants (554 males and 814 females: mean age of 68.8 years) of a health check-up examination. As previously reported, the demographic background of the subjects in this area is similar to that of the general Japanese population [15].

### Data collection

The subjects’ medical history, history of cardio-cerebrovascular diseases, use of alcohol, and smoking were ascertained by a questionnaire. Alcohol intake and smoking were classified as current habitual use or not. Height and weight were measured, and body mass index (BMI) was calculated as weight (kilograms) divided by the square of height (square meters) as an index of obesity. Waist circumference was measured at the level of the umbilicus in the standing position. Blood pressure (BP) was measured in the supine position twice at 3-min intervals using an upright standard sphygmomanometer. Vigorous physical activity and smoking were avoided for at least 30 min before BP measurement. The second BP with the fifth-phase diastolic pressure was used for analysis. Hypertensive subjects were defined as those with systolic BP ≥ 140 mmHg and/or those with diastolic BP ≥ 90 mmHg and/or those receiving antihypertensive medication. Subjects with fasting plasma glucose (FPG) ≥ 6.99 mmol/l (126 mg/dl) and/or subjects taking oral hypoglycemic agents or receiving insulin injection were diabetic. Subjects with dyslipidemia were defined as those with low-density lipoprotein cholesterol (LDL-c) ≥ 3.62 mmol/l (140 mg/dl) and/or triglycerides ≥ 1.69 mmol/l (150 mg/dl) and/or high-density lipoprotein cholesterol (HDL-c) < 1.03 mmol/l (40 mg/dl) and/or those taking lipid-lowering drugs.

Fasting blood samples were centrifuged within 1 h after collection. Serum levels of IGF-1 and GH were measured by ELISA and ECLIA methods to 1363 subjects who could receive blood testing. The blood was submitted to the commercially available laboratory (SRL Inc. Fukuoka, Japan), and the intra- and inter-assay coefficient of variations of IGF-1 and GH, respectively, at the laboratory that performed the assays was 2.56% and 0.75%, and 3.06% and 0.73% [16]. Liver enzymes (alanine aminotransferase [ALT], aspartate aminotransferase [AST], and γ-glutamyl transpeptidase [γ-GTP]) were also measured. Estimated glomerular filtration rate (eGFR) was calculated by the following estimation formula that has been recommended by the Japan Society of Nephrology: eGFR (ml/min/1.73^2^) = (194 × Scr^−1.094^ × age^−0.287^) × (0.739 for females) [17]. In addition to these blood testing, trace elements (calcium, magnesium, iron, zinc, and phosphorus) and Hs-CRP as an inflammation marker were measured. The participants underwent measurements of handgrip strength using a calibrated strain-gauged dynamometer and expressed in kilograms (kg). They were asked to squeeze maximally with the dominant hand. The score of the dominant hand was used for analysis. Walk speed was measured over a 5-m course, marked out on level ground. Participants were asked to walk the course at their usual walking pace from a standing start. The time taken to complete the course was timed using a digital stopwatch and recorded to the nearest tenth of a second. The fastest time was used to derive walk speed in meters per second. Their cognitive functions were evaluated by the mini-mental state examination (MMSE). MMSE is designed to quickly measure global cognitive functioning, temporal and spatial orientation, attention, immediate and short-term memory, language, praxis, and calculation. Scores ranged from 0 to 30, with higher scores indicating better cognitive performance.

Carotid intima-media thickness (c-IMT) of the common carotid artery was determined by using duplex ultrasonography (Sonosite “TITAN,” ALOKA) with a 10-MHz transducer in the supine position. Longitudinal B-mode images at the diastolic phase of the cardiac cycle were recorded by a single trained technician who was blinded to the subjects’ background. We measured the only far wall of c-IMT. The images were magnified and measured on the screen and printed with a high-resolution line recorder (LSR-100A, Toshiba). We measured c-IMT according to the originally described method published in circulation [18]. Briefly, the c-IMT defined by Pignoli et al. [18] was measured as the distance from the leading edge of the first echogenic line to the leading edge of the second echogenic line. The first line represented the lumen-intimal interface; the collagen-containing upper layer of the tunica adventitia formed the second line. At each longitudinal projection, the site of the greatest thickness, including plaque, was sought along the arterial walls nearest the skin and farthest from the skin from the common carotid artery to the internal carotid artery. Three determinations of c-IMT of one artery were conducted at the site of the greatest thickness and at 2 other points, 1 cm upstream and 1 cm downstream from this site. The averaged value among the 6 IMTs (3 from the left and 3 from the right) was used as the representative value for each individual.

This study was approved by the Tanushimaru branch of the Japan Medical Association and by the local mayor, as well as by the ethics committee of Kurume University School of Medicine. All the participants gave informed consent. The Research Ethics Committee of the Kurume University School of Medicine (Process numbers 09019/2018) approved the study in conformity with the principles embodied in the declaration of Helsinki.

### Statistical analysis

Because of their skewed distribution, natural logarithmic transformations were performed for the values of triglycerides, Hs-CRP, IGF-1, and GH. These values after the analysis using log (natural)-transformed values were presented in the original scale (Tables 1 and 2). In Tables 1 and 2, these values are presented as the geometric mean and range. Sex (men = 0, women = 1), smoking habits (non-smoker and former smoker = 0, current smoker = 1), alcohol intake (non-drinker and former drinker = 0, current drinker = 1), and medications for hypertension, dyslipidemia, and diabetes (no = 0, yes = 1) were used as dummy variables. The mean serum albumin levels were classified into the following 4 groups: G1 (3.2–3.9 mg/dL), G2 (4.0–4.3 mg/dL), G3 (4.4–4.6 mg/dL), and G4 (4.7–5.3 mg/dL). The mean parameters stratified by the 4 albumin levels groups were compared using an analysis of variance (ANOVA). Age and sex-adjusted means of parameters stratified by the 4 serum albumin groups were compared using an analysis of covariance (ANCOVA). For the categorical parameters, the *χ*^2^ test was used to test differences among groups. Univariate regression analysis was performed using a linear regression analysis. Using the significant factors detected by the univariate analysis, multiple stepwise regression analysis was performed. *P* values of < 0.05 were considered to indicate statistical significance. All of the statistical analyses were performed using the SAS software program (version 9.4, SAS Institute, Cary, NC, USA).

**Table 1:** Baseline characteristics stratified by serum albumin groups

Variables	G1 (lowest)	G2	G3	G4 (highest)	*P* for trend
Number	31	355	632	345	
Albumin range (g/dL)	3.2–3.9	4.0–4.3	4.4–4.6	4.7–5.3	
Albumin (g/dL)	3.8 ± 0.1	4.2 ± 0.1	4.5 ± 0.1	4.8 ± 0.1	< 0.001
Age (years)	79.8 ± 8.8	72.7 ± 11.4	67.8 ± 10.9	65.4 ± 10.5	< 0.001
Sex (%males)	15 (48.4)	148 (41.7)	251 (39.7)	136 (39.4)	0.722
Body mass index (kg/m^2^)	22.3 ± 3.4	23.2 ± 3.7	23.1 ± 3.4	22.9 ± 3.5	0.486
Systolic blood pressure (mmHg)	138 ± 24	136 ± 20	139 ± 21	141 ± 21	0.025
Diastolic blood pressure (mmHg)	71 ± 11	77 ± 11	80 ± 11	83 ± 12	< 0.001
Total cholesterol (mg/dL)	170 ± 31	199 ± 32	210 ± 36	221 ± 36	< 0.001
HDL-cholesterol (mg/dL)	52 ± 12	60 ± 15	65 ± 16	70 ± 20	< 0.001
LDL-cholesterol (mg/dL)	101 ± 27	119 ± 28	126 ± 31	131 ± 31	< 0.001
Triglycerides (mg/dL)^a^ (range)	95 (43–199)	105 (30–530)	104 (27–878)	108 (31–996)	0.467
AST (IU/L)	23.5 ± 7.5	23.8 ± 9.4	24.0 ± 10.5	25.2 ± 9.0	0.205
ALT (IU/L)	17.6 ± 10.2	18.9 ± 11.6	20.3 ± 11.5	23.4 ± 16.4	< 0.001
γ-GTP (IU/L)	26.6 ± 30.8	31.6 ± 46.0	31.4 ± 31.1	38.0 ± 38.1	0.036
eGFR (ml/min/1.73m^2^)	60.8 ± 23.8	66.3 ± 17.8	70.0 ± 15.7	71.9 ± 14.5	< 0.001
Uric acid (mg/dL)	5.2 ± 1.3	5.3 ± 1.4	5.3 ± 1.3	5.4 ± 1.4	0.415
HbA_1c_ (%) (NGSP)	5.8 ± 0.7	5.8 ± 0.6	5.7 ± 0.5	5.8 ± 0.6	0.248
Alcohol intake (%yes)	11 (35.5)	142 (40.0)	304 (48.1)	183 (53.0)	0.003
Current smoking (%yes)	0 (0.0)	36 (10.1)	57 (9.0)	48 (14.0)	0.022
Medications					
Hypertension (%yes)	14 (45.2)	142 (40.0)	230 (36.5)	122 (35.5)	0.455
Hyperlipidemia (%yes)	10 (32.3)	76 (21.7)	164 (26.4)	91 (26.8)	0.253
Diabetes (%yes)	5 (16.1)	34 (9.6)	59 (9.4)	40 (11.6)	0.471
MMSE (score)	26.6 ± 4.2	27.8 ± 2.8	28.5 ± 2.1	28.8 ± 1.8	< 0.001
Grip (kg)	24.2 ± 8.2	25.7 ± 8.9	28.5 ± 9.1	29.3 ± 9.2	< 0.001
Walk (sec)	6.1 ± 1.9	5.6 ± 1.6	5.1 ± 1.2	4.9 ± 1.1	< 0.001
Hs-CRP (mg/dL) ^a^(range)	0.129 (0.003–5.370)	0.064 (0.003–6.530)	0.045 (0.002–1.770)	0.039 (0.003–3.830)	< 0.001
Growth hormone (mg/dL) ^a^(range)	1.40 (0.12–8.63)	0.87 (0.03–18.5)	0.73 (0.03–12.7)	0.61 (0.03–13.7)	< 0.001
IGF-1 (mg/dL) ^a^(range)	80.8 (20–241)	88.8 (31–233)	96.8 (43–216)	102.3 (40–223)	< 0.001
Calcium (mg/dL)	9.0 ± 0.32	9.34 ± 0.32	9.56 ± 0.32	9.83 ± 0.30	< 0.001
Magnesium (mg/dL)	2.13 ± 0.21	2.19 ± 0.16	2.20 ± 0.16	2.25 ± 0.17	< 0.001
Iron (μg/dL)	72.5 ± 27.2	90.7 ± 33.4	99.9 ± 34.3	108.1 ± 35.2	< 0.001
Zinc (μg/dL)	60.8 ± 12.9	67.1 ± 10.6	73.8 ± 10.7	79.4 ± 12.2	< 0.001
Phosphorus (mg/dL)	3.37 ± 0.64	3.49 ± 0.51	3.51 ± 0.48	3.58 ± 0.45	0.025
Total IMT (mm)	0.82 ± 0.26	0.76 ± 0.28	0.71 ± 0.18	0.69 ± 0.16	< 0.001

Data are mean ± SD or range, unless otherwise indicated

*Abbreviations*: *HDL* high-density lipoprotein, *LDL* low-density lipoprotein, *AST* aspartate aminotransferase, *ALT* alanine aminotransferase, *γ-GTP* gamma glutamyl transferase, *eGFR* estimated glomerular filtration rate, *HbA*_*1c*_ glycosylated hemoglobin A_1c_, *MMSE* mini-mental state examination, *Hs-CRP* high-sensitivity C-reactive protein, *IGF-1* insulin-like growth factor 1, *IMT* intima-media thickness

^a^Log-transforms values were used for the analysis

**Table 2:** Age and sex-adjusted means of parameters stratified by serum albumin groups

Variables	G1 (lowest)	G2	G3	G4 (highest)	*P* for trend
Number	31	355	632	345	
Albumin (g/dL)	3.81 ± 0.02	4.21 ± 0.01	4.50 ± 0.01	4.80 ± 0.01	< 0.001
Body mass index (kg/m^2^)	22.5 ± 0.6	23.3 ± 0.2	23.0 ± 0.1	22.8 ± 0.2	0.290
Systolic blood pressure (mmHg)	131.5 ± 3.6	134.2 ± 1.0	139.9 ± 0.8	142.9 ± 1.1	< 0.001
Diastolic blood pressure (mmHg)	72.5 ± 2.0	77.5 ± 0.6	80.3 ± 0.4	82.8 ± 0.61	< 0.001
Total cholesterol (mg/dL)	176.1 ± 6.1	200.5 ± 1.8	209.0 ± 1.3	219.1 ± 1.8	< 0.001
HDL-cholesterol (mg/dL)	59 ± 16	64 ± 16	65 ± 16	69 ± 16	< 0.001
LDL-cholesterol (mg/dL)	105.8 ± 5.4	121.1 ± 1.6	125.1 ± 1.2	129.8 ± 1.6	< 0.001
Triglycerides (mg/dL)* (range)	96 (43–199)	105 (30–530)	104 (27–878)	108 (31–996)	0.512
AST (IU/L)	23.1 ± 1.8	23.6 ± 0.5	24.0 ± 0.4	25.3 ± 0.5	0.126
ALT (IU/L)	19.2 ± 2.3	19.6 ± 0.7	20.2 ± 0.5	22.8 ± 0.7	0.004
γ-GTP (IU/L)	29.3 ± 6.6	32.8 ± 1.9	31.2 ± 1.4	36.8 ± 2.0	0.131
eGFR (ml/min/1.73 m^2^)	67.4 ± 2.7	68.6 ± 0.8	69.5 ± 0.6	69.9 ± 0.8	0.622
Uric acid (mg/dL)	5.03 ± 0.22	5.27 ± 0.06	5.28 ± 0.05	5.44 ± 0.06	0.107
HbA_1c_ (%)(NGSP)	5.77 ± 0.10	5.73 ± 0.03	5.72 ± 0.02	5.78 ± 0.03	0.287
Alcohol intake (%yes)	11 (35.5)	142 (40.0)	304 (48.1)	183 (53.0)	0.218
Current smoking (%yes)	0 (0.0)	36 (10.1)	57 (9.0)	48 (14.0)	0.081
Medications					
Hypertension (%yes)	14 (45.2)	142 (40.0)	230 (36.5)	122 (35.5)	0.219
Hyperlipidemia (%yes)	10 (32.3)	76 (21.7)	164 (26.4)	91 (26.8)	0.008
Diabetes (%yes)	5 (16.1)	34 (9.6)	59 (9.4)	40 (11.6)	0.226
MMSE (score)	27.3 ± 0.4	28.1 ± 0.1	28.4 ± 0.1	28.6 ± 0.1	0.002
Grip (kg)	26.1 ± 1.0	26.8 ± 0.3	28.3 ± 0.2	28.4 ± 0.3	< 0.001
Walk (second)	5.6 ± 0.2	5.5 ± 0.1	5.1 ± 0.1	5.1 ± 0.1	< 0.001
Hs-CRP (mg/dL) ^a^(range)	0.116 (0.003–5.370)	0.063 (0.003–6.530)	0.045 (0.002–1.770)	0.040 (0.003–3.830)	< 0.001
Growth hormone (mg/dL) ^a^(range)	1.19 (0.12–8.63)	0.81 (0.03–18.5)	0.74 (0.03–12.7)	0.66 (0.03–13.7)	0.017
IGF-1 (mg/dL) ^a^(range)	82.4 (20–241)	86.1 (31–233)	92.0 (43–216)	98.4 (40–223)	< 0.001
Calcium (mg/dL)	8.98 ± 0.06	9.34 ± 0.01	9.56 ± 0.01	9.83 ± 0.02	< 0.001
Magnesium (mg/dL)	2.12 ± 0.03	2.18 ± 0.01	2.20 ± 0.01	2.26 ± 0.01	< 0.001
Iron (μg/dL)	71.2 ± 6.1	90.4 ± 1.8	100.0 ± 1.3	108.3 ± 1.8	< 0.001
Zinc (μg/dL)	61.0 ± 2.0	67.1 ± 0.6	73.8 ± 0.4	79.3 ± 0.6	< 0.001
Phosphorus (mg/dL)	3.45 ± 0.08	3.51 ± 0.02	3.50 ± 0.02	3.56 ± 0.02	0.241
Total IMT (mm)	0.75 ± 0.04	0.73 ± 0.01	0.71 ± 0.01	0.71 ± 0.01	0.239

Data are mean ± SD or range, unless otherwise indicated

*Abbreviations*: *HDL* high-density lipoprotein, *LDL* low-density lipoprotein, *AST* aspartate aminotransferase, *ALT* alanine aminotransferase, *γ-GTP* gamma glutamyl transferase, *eGFR* estimated glomerular filtration rate, *HbA*_*1c*_ glycosylated hemoglobin A_1c_, *MMSE* mini-mental state examination, *Hs-CRP* high-sensitivity C-reactive protein, *IGF-1* insulin-like growth factor 1, *IMT* intima-media thickness

^a^Log-transforms values were used for the analysis

## Results

Table 1 shows the characteristics of the 1363 subjects stratified by the 4 serum albumin groups. The characteristics of age (*p* < 0.001; inversely), systolic (*p* = 0.025), and diastolic (*p* < 0.001) BPs, total cholesterol (*p* < 0.001), HDL-c (*p* < 0.001), LDL-c (*p* < 0.001), ALT (*p* < 0.001), γ-GTP (*p* = 0.036), eGFR (*p* < 0.001), alcohol intake (*p* = 0.003), current smoking (*p* = 0.022), MMSE score (*p* < 0.001), handgrip strength (*p* < 0.001), times of walk 5 m (*p* < 0.001; inversely), Hs-CRP (*p* < 0.001; inversely), GH (*p* < 0.001; inversely), IGF-1 (*p* < 0.001), calcium (*p* < 0.001), magnesium (*p* < 0.001), iron (*p* < 0.001), zinc (*p* < 0.001), phosphorus (*p* = 0.025), and total IMT (*p* < 0.001; inversely) were significantly associated with the albumin quartiles.

Table 2 shows the age and sex-adjusted means of parameters stratified by the 4 serum albumin groups. The characteristics of systolic (*p* < 0.001) and diastolic (*p* < 0.001) BPs, total cholesterol (*p* < 0.001), HDL-c (*p* < 0.001), LDL-c (*p* < 0.001), ALT (*p* = 0.004), medication for hyperlipidemia (*p* = 0.008), MMSE score (*p* = 0.002), handgrip strength (*p* < 0.001), times of walk 5 m (*p* < 0.001; inversely), Hs-CRP (*p* < 0.001; inversely), GH (*p* = 0.017; inversely), IGF-1 (*p* < 0.001), calcium (*p* < 0.001), magnesium (*p* < 0.001), iron (*p* < 0.001), and zinc (*p* < 0.001) were significantly associated with the 4 albumin groups.

Table 3 shows the results of univariate analyses for correlates of serum albumin levels. Age (*p* < 0.001), systolic (*p* < 0.001), and diastolic (*p* < 0.001) BPs, total cholesterol (*p* < 0.001), HDL-c (*p* < 0.001), LDL-c (*p* < 0.001), triglycerides (*p* = 0.011), AST (*p* < 0.001), ALT (*p* < 0.001), γ-GTP (*p* = 0.011), eGFR (*p* < 0.001), alcohol intake (*p* < 0.001), current smoking (*p* = 0.020), MMSE score (*p* < 0.001), handgrip strength (*p* < 0.001), times of walk 5 m (*p* < 0.001; inversely), Hs-CRP (*p* < 0.001; inversely), GH (*p* < 0.001; inversely), IGF-1 (*p* < 0.001), calcium (*p* < 0.001), magnesium (*p* < 0.001), iron (*p* < 0.001), zinc (*p* < 0.001), phosphorus (*p* = 0.002), and total IMT (*p* < 0.001; inversely) were significantly associated with albumin levels.

**Table 3:** Univariate analysis for serum albumin

Variables	*β*	SE	*p*
Age (years)	− 0.007	0.001	< 0.001
Sex (%males)	0.006	0.014	0.689
Body mass index (kg/m^2^)	0.001	0.002	0.792
Systolic blood pressure (mmHg)	0.001	0.001	< 0.001
Diastolic blood pressure (mmHg)	0.006	0.001	< 0.001
Total cholesterol (mg/dL)	0.002	0.001	< 0.001
HDL-cholesterol (mg/dL)	0.004	0.001	< 0.001
LDL-cholesterol (mg/dL)	0.002	0.001	< 0.001
Triglycerides (mg/dL)^a^	0.034	0.014	0.011
AST (IU/L)	0.002	0.001	< 0.001
ALT (IU/L)	0.003	0.001	< 0.001
γ-GTP (IU/L)	0.0004	0.0002	0.011
eGFR (ml/min/1.73m^2^)	0.002	0.001	< 0.001
Uric acid (mg/dL)	0.009	0.005	0.107
HbA_1c_ (%) (NGSP)	− 0.003	0.013	0.829
Alcohol intake (%yes)	0.057	0.014	< 0.001
Current smoking (%yes)	0.053	0.023	0.020
Medication			
Hypertension (%yes)	− 0.019	0.015	0.202
Hyperlipidemia (%yes)	0.020	0.016	0.212
Diabetes (%yes)	0.017	0.023	0.457
MMSE (score)	0.021	0.003	< 0.001
Grip (kg)	0.005	0.001	< 0.001
Walk (sec)	− 0.043	0.005	< 0.001
Hs-CRP (mg/dL)^a^	− 0.041	0.006	< 0.001
Growth hormone (mg/dL)^a^	− 0.032	0.006	< 0.001
IGF-1 (mg/dL)^a^	0.237	0.021	< 0.001
Calcium (mg/dL)	0.401	0.015	< 0.001
Magnesium (mg/dL)	0.279	0.041	< 0.001
Iron (μg/dL)	0.002	0.001	< 0.001
Zinc (μg/dL)	0.009	0.001	< 0.001
Phosphorus (mg/dL)	0.046	0.014	0.002
Total IMT (mm)	− 0.172	0.033	< 0.001

*Abbreviations*: *HDL* high-density lipoprotein, *LDL* low-density lipoprotein, *AST* aspartate aminotransferase, *ALT* alanine aminotransferase, *γ-GTP* gamma glutamyl transferase, *eGFR* estimated glomerular filtration rate, *HbA*_*1c*_ glycosylated hemoglobin A_1c_, *MMSE* mini-mental state examination, *Hs-CRP* high-sensitivity C-reactive protein, *IGF-1* insulin-like growth factor 1, *IMT* intima-media thickness, *SE* standard error

^a^Log-transforms values were used for the analysis

Using the significant factors detected by univariate analysis in Table 3, we performed the multiple stepwise regression analysis (Table 4). Eventually, the significances of age (*p* < 0.001), calcium (*p* < 0.001), zinc (*p* < 0.001), magnesium (*p* < 0.001), iron (*p* < 0.001), Hs-CRP (*p* < 0.001; inversely), systolic BP (*p* < 0.001), GH (*p* < 0.001; inversely), IGF-1 (*p* = 0.004), eGFR (*p* = 0.007), handgrip strength (*p* = 0.013), and MMSE (*p* = 0.024) were still remained.

**Table 4:** Multiple stepwise regression analysis for serum albumin

Variables	*β*	SE	*p*
Calcium (mg/dL)	0.336	0.015	< 0.001
Zinc (μg/dL)	0.005	0.0004	< 0.001
Age	− 0.003	0.0006	< 0.001
Magnesium (mg/dL)	0.206	0.030	< 0.001
Systolic blood pressure (mmHg)	0.001	0.0002	< 0.001
Hs-CRP (mg/dL)^a^	− 0.046	0.013	< 0.001
Growth hormone (mg/dL)^a^	− 0.008	0.004	< 0.001
Iron (μg/dL)	0.001	0.003	< 0.001
IGF-1 (mg/dL) ^a^	0.0003	0.0002	0.004
eGFR (ml/min/1.73 m^2^)	0.001	0.0003	0.007
Grip (kg)	0.002	0.001	0.013
MMSE (score)	0.005	0.002	0.024

*Abbreviations*: *Hs-CRP* high-sensitivity C-reactive protein, *IGF-1* insulin-like growth factor 1, *eGFR* estimated glomerular filtration rate, *MMSE* mini-mental state examination, *SE* standard error

*R*^2^ = 0.53

^a^Log-transforms values were used for the analysis

## Discussion

The novel finding of the present study was that the lower albumin level, even in the normal range, was strongly associated with as frailty measures, trace elements, and an inflammation marker in a general population, independent of age.

### Lower albumin levels and frailty measures

We found that handgrip strength, times of walk 5 m, GH, and IGF-1 were significantly associated with the 4 albumin groups. Reports from Smit et al. [6] suggested that serum albumin levels were lower in frail US adults. They defined frailty based on the four domains by Fried et al. [19] as follows: (1) slow walking, (2) muscle weakness, (3) exhaustion, and (4) low physical activity. Lower serum albumin may have been shown to be associated with longer times of walk followed by greater loss of muscle mass and may be an early indicator of impending muscle strength decline as suggested by Schalk et al. [20].

There are many causes of frailty. Of these, alterations in hormones represent major factors involved in the pathophysiology of frailty [21]. The clear-cut effects of GH and IGF-1 on serum albumin levels in healthy individuals have not been demonstrated. Especially, although the effects of GH on bone and muscle in GH-replete individuals have been studied [22], further investigation remained to be required. Our data in the multiple stepwise regression analyses showed that GH is inversely associated with albumin levels, which can be interpreted by the fact that the declining activity of GH with age in part responsible for better nutritional status [23]. In the final analysis, serum IGF-1 is positively associated with albumin levels. As recent epidemiological survey supported our study [24], it was indicated that lower IGF-1 levels were associated with lower handgrip strength and worse physical performance. This may be also because human aging is accompanied by a decrease in serum IGF-1 secretion.

Cognitive dysfunction is one of the important factors of frailty measures. Our study showed that MMSE score was significantly and independently associated with serum albumin. Ng et al. reported low albumin was an independent risk marker for cognitive decline in community-living 1664 Chinese older adults [25]. Although the data is limited to elderly hip fracture patients, lower serum albumin levels are independently associated with lower MMSE scores [26].

### Lower albumin levels and trace elements

Of trace elements we have measured, calcium, zinc, magnesium, and iron were positively and significantly associated with albumin levels in the final stepwise regression analysis. It is well known that calcium and iron are closely associated with human nutritional status [27]. On the other hand, Peng et al. [8] suggested serum zinc was a significant independent risk factor for hypoalbuminemia and hospital admissions. Ongan et al. [28] also reported that iron, zinc, and magnesium intake of malnourished elderly persons was lower. Recently, Japanese investigators have suggested that zinc intake was associated with mental disorder, whereas calcium, magnesium, and iron intake were not associated with mental disorders [29]. The impact of dietary mineral intake on mental health is still controversial.

### Lower albumin levels and an inflammation marker

The present study revealed that lower albumin levels are strongly associated with an inflammatory marker such as Hs-CRP. Hypoalbuminemia, which can be associated with various diseases, is frequently observed in dialysis patients, as previously reported [30, 31]. One suggested that levels of inflammatory biomarkers such as CRP and interleukin-6 are increased in hypoalbuminemic compared with normoalbuminemic dialysis patients [30]. The other suggested that hypoalbuminemia and inflammation may play an important role in atherogenesis [31].

Our previous study suggested that lower albumin levels have a strong impact on cerebro-cardiovascular mortality [3]. The enrolled subjects were free from apparent cerebro-cardiovascular disease at baseline, and their serum albumin levels were within the normal range; however, lower albumin levels and Hs-CRP can affect the high mortality on future cerebro-cardiovascular events in this population, because the inflammation is one of the important risk factors for atherosclerosis [32]. The precise mechanism underlying the relationship between lower albumin and an inflammation marker should be clarified in future studies.

Clinical roles of albumin are frequently discussed in hospitalized patients [33–35]. However, there are few reports on the association between lower albumin levels and related factors in a general population. In the clinical practice, lower albumin levels [36] are commonly discovered in association with nutritional deterioration and disease-related inflammatory response. Along with the evolution of the disease itself, this condition might be a result of the aging process, with levels of albumin decreasing with advancing age. However, the association between the lower albumin levels and age has not been fully elucidated; therefore, the association should also take into account diseases and other age-related conditions rather than age alone.

### Limitations

The limitations of this study were as follows. First, because our study design is cross-sectional, we cannot clarify the changes of time trend in albumin. Prospective studies are planned to investigate the role of the factors in the subjects with lower albumin levels in our future studies after long-term follow-up. Second, we performed only a single blood testing to evaluate an association between lower albumin and related parameters. The quadrant shift may occur in the same subjects. Third, the pathophysiological mechanism underlying the association between low albumin levels and related factors was not revealed from our observational study. Nevertheless, clear positive or inverse relationships between lower albumin levels and related factors were striking and further investigation should be required. Fourth, we have no data regarding gastrointestinal tract function in this study.

## Conclusions

Lower albumin levels were found to be related factors of frailty measures, trace elements, and an inflammation marker in a general population.

## Abbreviations

MMSE: Mini-mental state examination
eGFR: Estimated glomerular filtration rate
IMT: Intima-media thickness
GH: Growth hormone
IGF-1: Insulin-like growth factor-1
Hs-CRP: High-sensitivity C-reactive protein
BMI: Body mass index
BP: Blood pressure
FPG: Fasting plasma glucose
LDL-c: Low-density lipoprotein cholesterol
HDL-c: High-density lipoprotein cholesterol
